# Predictive value of subacromial motion metrics for the effectiveness of ultrasound-guided dual-target injection: a longitudinal follow-up cohort trial

**DOI:** 10.1186/s13244-025-01989-5

**Published:** 2025-07-01

**Authors:** Wei-Ting Wu, Che-Yu Lin, Yi-Chung Shu, Lan-Rong Chen, Levent Özçakar, Ke-Vin Chang

**Affiliations:** 1 Department of Physical Medicine and Rehabilitation and Community and Geriatric Research Center, National Taiwan University Hospital, Bei-Hu Branch, Taipei City, Taiwan; 2 Department of Physical Medicine and Rehabilitation, National Taiwan University College of Medicine, Taipei, Taiwan; 3 Institute of Applied Mechanics, College of Engineering, National Taiwan University, Taipei, Taiwan; 4 Department of Physical and Rehabilitation Medicine, Hacettepe University Medical School, Ankara, Turkey; 5 Center for Regional Anesthesia and Pain Medicine, Wang-Fang Hospital, Taipei Medical University, Taipei, Taiwan

**Keywords:** Rehabilitation, Shoulder impingement syndrome, Corticosteroid, Ultrasound, Rotator interval

## Abstract

**Objective:**

Subacromial impingement syndrome (SIS) frequently causes shoulder pain. This study aimed to (1) assess the predictive utility of quantitative dynamic subacromial ultrasound for ultrasound-guided dual-target injections and (2) compare the long-term efficacy of dual-target injections with standard subdeltoid-subacromial injections in SIS patients.

**Methods:**

Patients with SIS received 40 mg of triamcinolone acetonide via ultrasound-guided dual-target injections (subdeltoid-subacromial bursa and long head of the biceps brachii tendon). Clinical assessments and static/dynamic ultrasound were performed at baseline and 4 weeks post-procedure. Minimal vertical acromiohumeral distance (mVAHD) was measured by tracing the humeral greater tuberosity against the acromion. A historical cohort receiving standard subdeltoid-subacromial corticosteroid injections was used for comparison.

**Results:**

Of 90 patients receiving dual-target injections, 70 (77.7%) achieved early treatment success. An enlarged minimal mVAHD was associated with success, except during the abduction phase in the full-can posture. Among these 70 patients, 25 (35.7%) had shoulder pain recurrence requiring repeat injections, linked to a decreased mVAHD across all phases and postures. Compared to 90 patients in a historical cohort receiving standard subdeltoid-subacromial injections, the dual-target group had a significantly longer mean time to pain recurrence (309.1 ± 130.1 days vs. 267.5 ± 184.2 days, *p* = 0.03).

**Conclusion:**

Dynamic ultrasound metrics, including mVAHD, predict early success and pain recurrence following dual-target injections in SIS. Dual-target injections offer a longer duration of effectiveness compared to standard subdeltoid-subacromial injections. Future research should explore the predictive value of mVAHD with deep learning algorithms and evaluate the approach in adhesive capsulitis.

**Trial registration:**

ClinicalTrials.gov (NCT04219527). Registered on 27 December 2019, https://clinicaltrials.gov/study/NCT04219527.

**Critical relevance statement:**

Dynamic ultrasound metrics predict early success and pain recurrence following dual-target injections in SIS, offering a longer duration of effectiveness compared to standard subdeltoid-subacromial injections.

**Key Points:**

- Dynamic ultrasound metrics predict injection success and pain recurrence in impingement.
- Dual-target injections offer a longer duration of effectiveness than standard injections.
- Future research should assess deep learning’s predictive value in adhesive capsulitis.

**Graphical Abstract:**

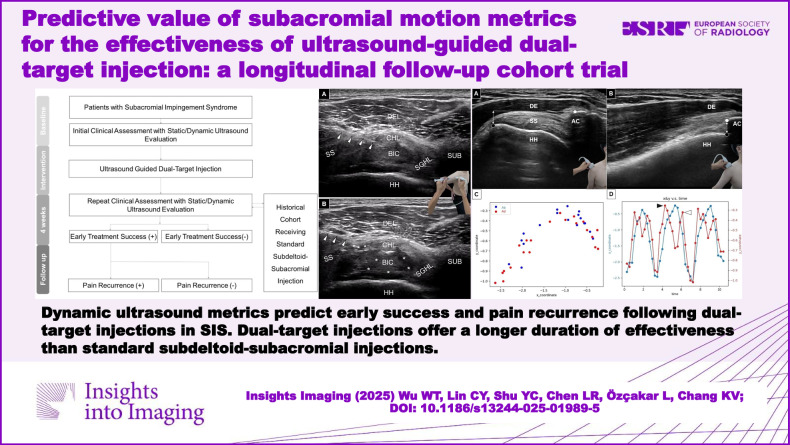

## Introduction

Shoulder pain is a common musculoskeletal disorder with a median community prevalence of 16% (ranging from 0.67 to 55.2%) according to a systematic review, with subacromial impingement syndrome (SIS) accounting for 44 to 65% of shoulder complaints [1]. This syndrome arises from extrinsic compression due to limited space between the humeral head and the anterior acromion, coracohumeral ligament, and acromioclavicular joint, as well as intrinsic degradation of the rotator cuff through diminished vascular supply and tensile forces [2]. Corticosteroid injection is one of the most effective non-surgical management options. However, Marks et al pointed out that longer symptom duration and higher age are significantly associated with poorer outcomes following palpation-guided subacromial injection [3].

Ultrasound guidance has become mainstream for musculoskeletal injections due to its superior resolution on soft tissue and non-radiation benefits. A meta-analysis highlighted the potential benefit of ultrasound-guided subacromial injection over anatomy-guided methods for clinical symptom improvement [4]. While ultrasound imaging excels in delineating rotator cuff pathologies, Bouju et al [5] revealed no correlation between static ultrasonography findings and the efficacy of a local anesthetic injection into the subacromial bursa. On the other hand, Chang et al [6] reported the potential benefit of dynamic ultrasound in predicting the effect of standard ultrasound-guided subacromial injection, revealing that grade 2 subacromial impingement during dynamic ultrasound examination is a prognostic factor for initial effectiveness. In recent years, dynamic subacromial ultrasound imaging has evolved from qualitative evaluation to quantitative analysis [7]. However, no study has yet examined the effectiveness of quantitative subacromial ultrasound assessment in predicting the success of subdeltoid-subacromial injections.

Although ultrasound-guided subdeltoid-subacromial injections relieve SIS early, recurrent pain persists as they do not address glenohumeral capsule issues. In 2019, Chang et al developed a dual-target injection technique, administering 40 mg triamcinolone acetonide to both the subacromial-subdeltoid bursa and the glenohumeral joint [8]. This method showed less rebounding pain at the 3-month follow-up compared to standard subacromial injections, but its long-term effects (> 6 months) are unverified. This study aims to (1) assess the utility of quantitative dynamic subacromial ultrasound in predicting the effectiveness of ultrasound-guided dual-target injections, and (2) evaluate the long-term efficacy of dual-target injections compared with standard subdeltoid-subacromial injection. We hypothesize that dynamic subacromial ultrasound can predict the superior effectiveness of dual-target injections over standard injections.

## Methods

### Participant characteristics

**Fig. 1:**
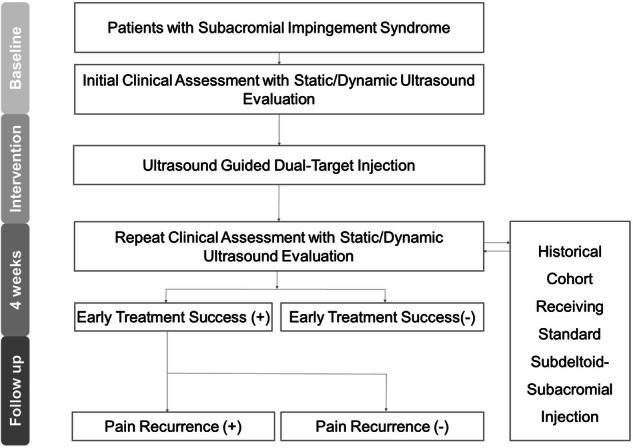
Flow diagram of participant recruitment, clinical and ultrasound assessments, follow-up before and after dual-target injection, and comparison with a historical cohort

Adults over 20 years old with shoulder pain persisting for more than 3 months were recruited from the physical medicine and rehabilitation clinic. Informed consent was obtained before participation, and the research project was approved by the institutional review board (IRB No. 201910036RINC). SIS was diagnosed based on at least one positive result from the Neer impingement test, Hawkins-Kennedy impingement test, or painful arc test [8, 9]. Additional inclusion criteria included (1) ultrasound imaging identifying subdeltoid bursitis on static ultrasound findings or at least grade-1 subacromial impingement on dynamic images (pain during shoulder elevation without soft tissue impingement), (2) a lack of response to oral analgesics and physical therapy, and (3) no limitations in passive shoulder range of motion. Participants were excluded if they had significant trauma or recent injections within 6 months before enrollment in the impacted shoulder, a history of intolerance to corticosteroid or lidocaine, upper limb pain not originating from the shoulder, or a history of cancer and systemic rheumatic diseases (such as rheumatoid arthritis). The research flow diagram is shown in Fig. 1.

### Determination of shoulder pathologies

Before the intervention, each involved shoulder was scrutinized by ultrasound using the EURO-MUSCULUS/USPRM scanning protocol [10, 11]. Peritendinous effusion over 1 mm in thickness around the long head of the biceps brachii tendon was deemed pathological [12]. Subdeltoid bursitis was confirmed by measuring the bursa thickness to be greater than 2 mm [6]. Hyperechoic plaques within the tendon, accompanied by acoustic shadows, were indicative of intra-tendinous calcification. Rotator cuff tears were identified by visible separations or absence of tendon tissue, classified as full-thickness or partial-thickness. Full-thickness tears extended from the subdeltoid bursa to the humeral head, while partial-thickness tears did not span the entire tendon.

### Subacromial motion metrics

The study used a linear ultrasound transducer (5–18 MHz; HI VISION Ascendus, Hitachi) for assessment. The transducer was positioned along the scapular plane, with its midpoint aligned with the lateral edge of the acromion to ensure clear visualization of the humeral head, supraspinatus tendon, and acromion. The scanning depth was set to 30 mm, with the focus at 20 mm, and the frame rate at 300 frames per second. The gain and dynamic range of the ultrasound machine were kept constant throughout the examination. The greater tuberosity, supraspinatus tendon, and acromion were clearly visible on the ultrasound monitor. A musculoskeletal physician with over 10 years of experience in shoulder ultrasound diagnosis conducted both static and dynamic ultrasound imaging, followed by guided dual-target intervention for all participants.

**Fig. 2:**
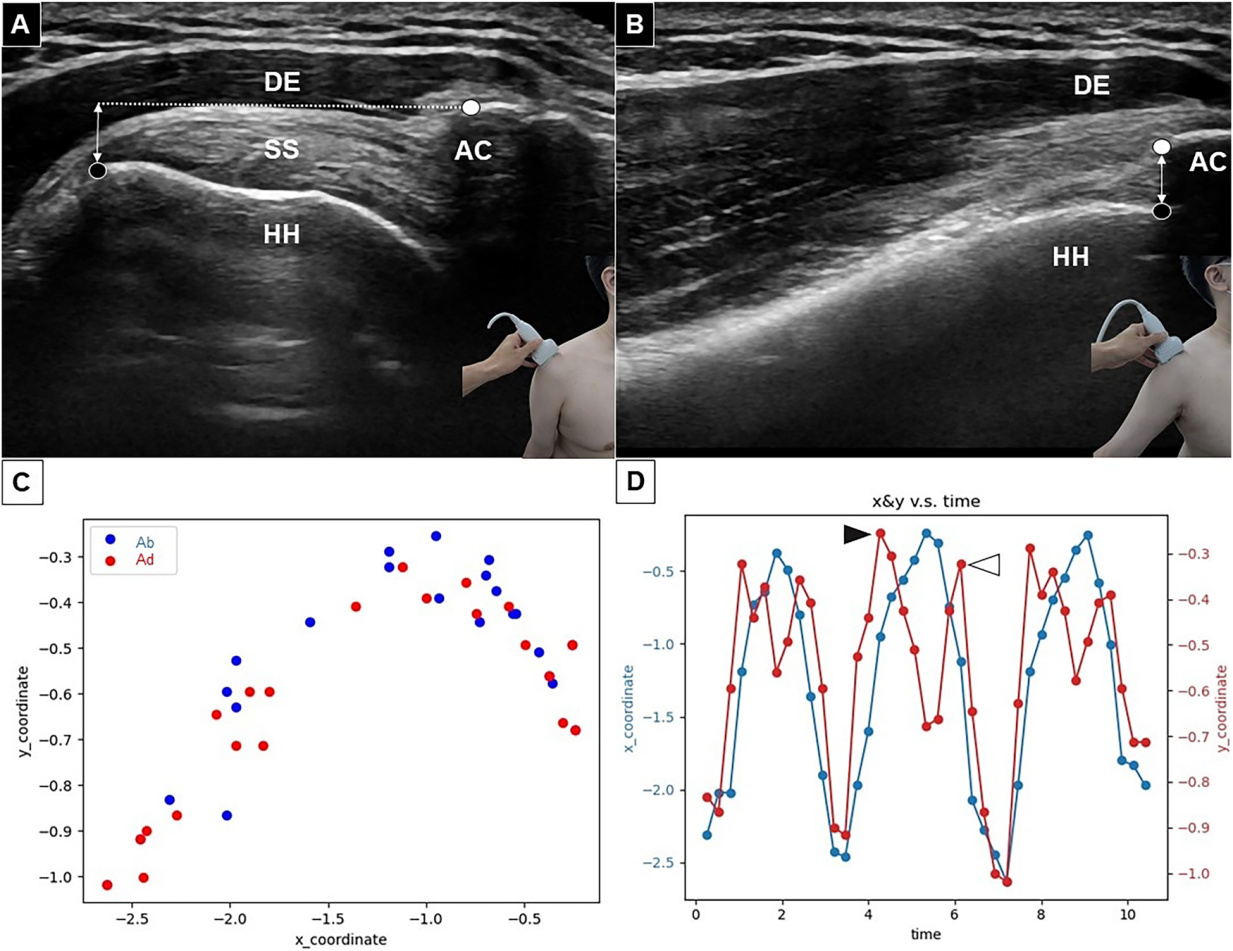
Dynamic subacromial imaging is shown in the starting (**A**) and abducted (**B**) positions. The dashed line indicates the horizontal plane passing through the lateral edge of the acromion, while the double-headed line represents the vertical acromiohumeral distance. The black dot marks the greater tuberosity of the humerus, and the white dot marks the lateral edge of the humerus. The relative positions of the greater tuberosity and the lateral edge of the acromion are displayed on a two-dimensional plot (**C**). The coordinates of the greater tuberosity along the *X*- and *Y*-axes are plotted over time (**D**). The black arrowhead indicates the minimal vertical acromiohumeral distance during abduction, while the white arrowhead shows the minimal vertical acromiohumeral distance during adduction. The blue line and its points represent the trajectory of the greater tubercle’s *X*-coordinate relative to the lateral acromion, while the red line and its points represent the trajectory of its *Y*-coordinate

Participants were instructed to abduct their arms until the greater tuberosity rotated beneath the acromion (Fig. 2A), then return to the neutral position repeatedly (Fig. 2B). The arm movement speed was similar to that used when reaching for an overhead object [7].

The dynamic video clips were revised to include three motion cycles with clear visualization of the lateral acromial edge and greater tuberosity for analysis. The video was output as images at four frames per second, with each labeled at the lateral acromion and greater tuberosity. If the greater tuberosity was unclear, the most prominent point of the humeral head was used instead. The trajectory of the greater tuberosity under the acromion was calculated by plotting its movement relative to the acromion’s lateral edge on the *X* and *Y*-axes (Fig. 2C). In the time-location curve on the *Y*-axis, each peak represented when the greater tuberosity rotated beneath the acromion, and each trough indicated its return to the initial position. The minimal vertical acromiohumeral distance (mVAHD) was identified at different phases across the three cycles for analysis (Fig. 2D). A previous dynamic ultrasound study [7] demonstrated high intra-rater and inter-rater reliabilities for the measurement of mVAHD, with intraclass correlation coefficients of 0.94 and 0.88, respectively.

### Ultrasound-guided injection technique

**Fig. 3:**
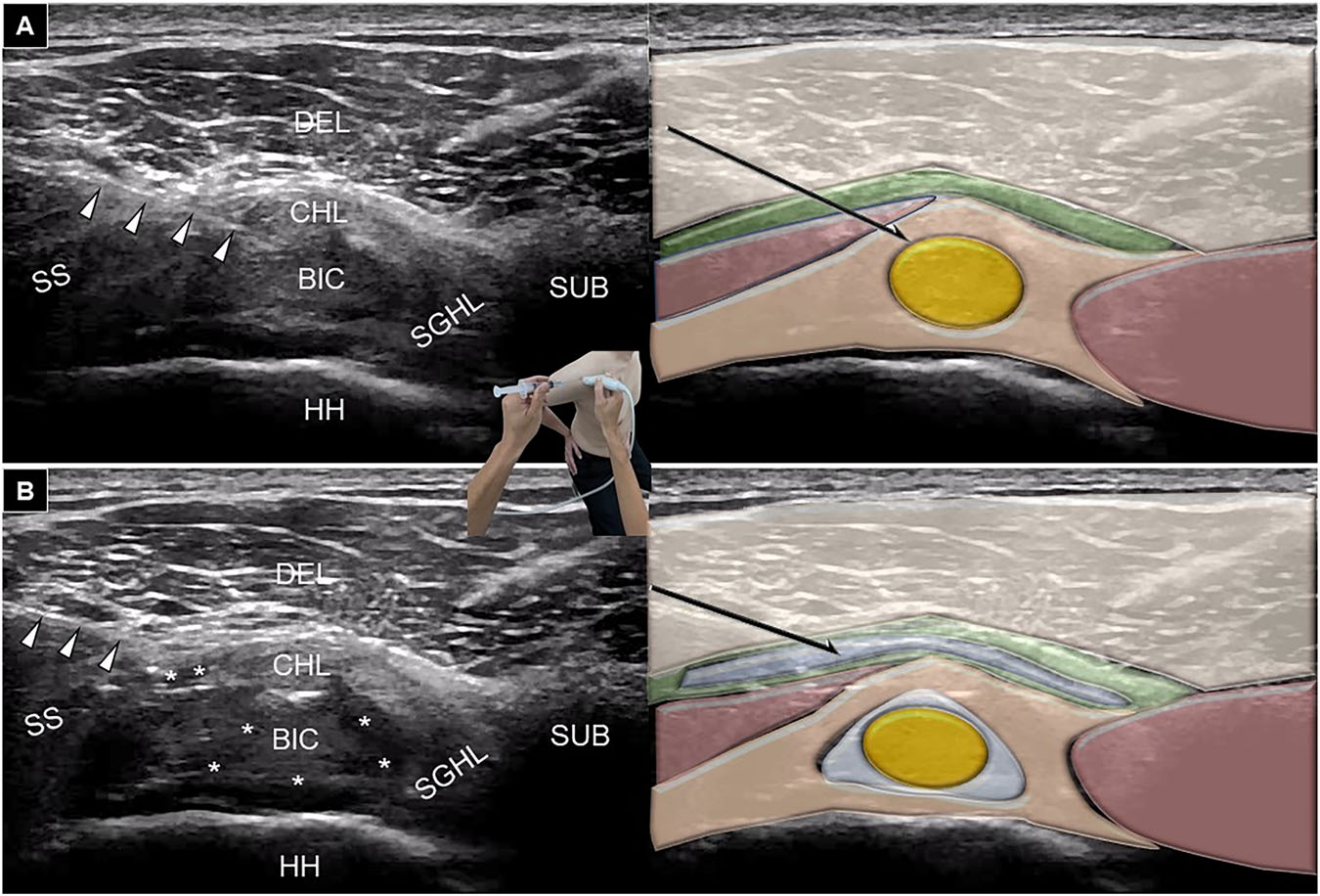
Ultrasound imaging (left) and schematic drawing (right) showing a dual-target injection, with the needle tip initially positioned in the peritendinous region of the long head of the biceps brachii tendon (**A**), followed by retraction to reach the subdeltoid-subacromial bursa (**B**). SS, supraspinatus tendon; DE, deltoid muscle; CHL, coracohumeral ligament; BIC, long head of the biceps brachii tendon; HH, humeral head; SGHL, superior glenohumeral ligament; SUB, subscapularis tendon. Asterisks: injectate; white arrowheads: needle

Patients were positioned with arms extended and hands on the affected side’s buttock, using the modified Crass maneuver. The transducer was placed on the supraspinatus tendon in the axial view near the rotator cuff interval. For the dual-target injection, the physician inserted the needle from lateral to medial to target the rotator cuff interval [8]. A previous cadaveric study has indicated that the lateral-to-medial approach achieves better intra-articular spread than the medial-to-lateral approach [13]. The peritendinous space of the long head of the biceps brachii tendon was injected before the subacromial-subdeltoid bursa to prevent the crystalloid corticosteroid from obscuring the needle. The injectant, containing 40 mg triamcinolone acetonide and 3 mL of 1% lidocaine, was administered in two stages. Upon injection, the coracohumeral ligament was first pierced, followed by entering the sheath of the long head of the biceps brachii tendon, and administering 2 milliliters of the injectate (Fig. 3A). Subsequently, the needle was withdrawn to the subdeltoid-subacromial bursa where the remaining 2 milliliters of the injectant were delivered (Fig. 3B). In the standard subdeltoid-subacromial injection group, the needle was likewise inserted from lateral to medial to access the subdeltoid-subacromial bursa [6], and the identical mixture and volume of injectant as used in the dual-target injection were then fully administered into the bursa.

### Clinical outcome assessment

A thorough physical examination was performed, including tests for bicipital groove tenderness, Speed’s test, Yergason’s test, the empty can test, Neer’s test, Hawkins-Kennedy test, and the painful arc test. The primary outcome was measured using the visual analog scale (VAS) for pain (at rest, during the night, and during overhead activities), whereas the Chinese version of the Shoulder Pain and Disability Index (SPADI) was treated as the secondary outcome [13]. Regarding the reliability of the VAS for musculoskeletal pain, Boonstra et al [14] reported reliability coefficients ranging from 0.60 to 0.77. For the SPADI, Roach et al [15] demonstrated test-retest reliability ranging from 0.6377 to 0.6552, with internal consistency between 0.8604 and 0.9507. The VAS, ranging from 0 to 10 cm, quantified pain intensity [14]. The SPADI consists of 13 items evaluating shoulder pain and function, with each item rated from 0 (no pain or no difficulty) to 10 (worst pain or extreme difficulty). These scores are then converted to a 100-point scale. All clinical assessments were performed by a research assistant.

All patients were scheduled for a follow-up visit 4 weeks after the procedure. A successful early outcome was defined as a reduction of over 30% in VAS scores in any of the three pain subdomains [16]. After the 4-week follow-up, patients were not advised to return to the clinic unless they experienced a recurrence of symptoms [6]. Recurrence was defined as the need for a subsequent injection due to recurrent shoulder pain in patients who initially responded to treatment [6]. Patients who visited any department in our hospital for at least 365 days post-injection without documented shoulder complaints requiring treatment were designated as “no recurrence.” This 365-day period was based on the time between the last dual-target injection and data retrieval. Through our electronic chart system, we reviewed all inpatient and outpatient records of the enrolled patients. We verified all medical records following the day of enrollment, not limited to those from the department of physical medicine and rehabilitation, to confirm that patients classified as having “no recurrence” had no relevant musculoskeletal complaints related to the shoulders. The recruitment and follow-up time window is detailed in Supplementary Table 1.

A research assistant contacted participants who had not visited any department within 365 days to confirm if their shoulder pain had resolved or if they received another injection elsewhere. If another injection was reported, they were classified as “recurrence.” Participants who did not respond to follow-up calls were censored in the survival analysis. Any recurrence requiring a second injection after 365 days was still labeled as “non-recurrence.”

To compare the long-term effects of the dual-target injection with those of the standard ultrasound-guided subdeltoid-subacromial corticosteroid injection, we retrospectively selected a historical cohort from the hospital’s clinical registry. Patients were matched by age within a 5-year range and had to have a documented diagnosis of SIS in their electronic medical records. The recurrence definitions, follow-up protocols, and observation periods were identical to those used in the dual-target injection group.

### Statistical analysis

Continuous variables are presented as means with standard deviations, and their distributions were assessed using the Shapiro-Wilk test [17]. For normally distributed data, comparisons were made using an independent Student’s *t*-test. For non-normally distributed data, the Mann–Whitney U test was employed [18]. Categorical variables are shown as counts and percentages. The Chi-square test or Fisher’s exact test (for sparse data) was used to compare proportions between groups. The Wilcoxon signed-rank test [19] was used to compare paired data when assessing the difference between two related samples or repeated measurements on a single sample. Statistical significance was determined at a *p*-value of less than 0.05.

Logistic regression analysis was conducted to evaluate the associations between early treatment success and static/dynamic sonographic findings, focusing on items statistically significant in the univariate analysis [20]. Results are presented as odds ratios (OR) with 95% confidence intervals (CI). The analysis was adjusted for age, gender, and affected shoulder sides. A Cox proportional hazards model [21] was employed to predict the recurrence of shoulder pain following injection, with recurrence defined as the necessity of a second injection. The primary predictor in this model was mVAHD, derived from subacromial motion metrics and adjusted for age, gender, and the affected shoulder side. The results are presented as hazard ratios (HR) with corresponding 95% confidence intervals (CI). Receiver operating characteristic (ROC) analysis was used to identify the most informative cut-off point of the mVAHD to predict either early treatment success or recurrence of shoulder pain, using the Youden index, calculated as Sensitivity + Specificity − 1, to determine the optimal cut-off point with the highest value designated as the best cut-off value [22]. The log-rank test was used to compare the time to pain recurrence between the group receiving dual-target injections and the group undergoing standard subacromial injections [23]. A *p*-value below 0.05 was considered statistically significant, and statistical analysis was performed using SPSS version 21.0 (IBM SPSS Statistics for Windows).

## Results

### Participant characteristics

A total of 90 patients (56 females and 34 males) with unilateral shoulder pain were enrolled from October 2020 to November 2022 for dual-target injections, without loss to follow-up at the 4-week post-injection mark. Their average age was 59.8 ± 10.4 years. The baseline VAS in cm for pain was 6.2 ± 0.9 at rest, 6.3 ± 1.0 at night, and 6.5 ± 1.0 during overhead activity, which decreased to 2.8 ± 1.5, 2.8 ± 1.5, and 2.8 ± 1.6, respectively, at 4 weeks post-injection. The SPADI was 62.3 ± 20.7 at baseline and decreased to 29.7 ± 23.0 following the injection. At baseline, static ultrasound imaging revealed a higher percentage of biceps tenosynovitis, supraspinatus tendinopathy, and infraspinatus tendinopathy on the painful side compared to the asymptomatic side. Additionally, while the mVAHD on the painful side tended to be smaller than on the asymptomatic side, this difference did not reach statistical significance (Table 1).

**Table 1:** Static sonographic findings and minimal vertical acromiohumeral distance of patients’ bilateral shoulders before dual-target injection

	Patients (*n* = 90)		
	Non-affected side	Affected side	*p*-value
Static sonographic findings			
Biceps tenosynovititis	14 (15.6%)	33 (36.7%)	0.001*
Subscapularis calcification	8 (8.9%)	10 (11.1%)	0.774
Subscapularis tear	1 (1.1%)	3 (3.3%)	0.500
Subscapularis tendinopathy	9 (10.0%)	15 (16.7%)	0.238
Supraspinatus calcification	9 (10.0%)	9 (10.0%)	1.000
Supraspinatus full-thickness tear	2 (2.2%)	5 (5.6%)	0.375
Supraspinatus partial tear	5 (5.6%)	6 (6.7%)	1.000
Supraspinatus tendinopathy	26 (28.9%)	40 (44.4%)	0.020*
Subdeltoid bursitis	6 (6.7%)	14 (15.6%)	0.115
Infraspinatus calcification	0 (0.0%)	5 (5.6%)	0.063
Infraspinatus tear	1 (1.1%)	0 (0.0%)	1.000
Infraspinatus tendinopathy	1 (1.1%)	7 (7.8%)	0.031*
Minimal vertical acromiohumeral distance (cm) at baseline			
Minimal vertical acromiohumeral distance (cm) Fab	0.32 ± 0.17 (0.28 to 0.36)	0.29 ± 0.18 (0.26 to 0.33)	0.278
Minimal vertical acromiohumeral distance (cm) Fad	0.35 ± 0.17 (0.32 to 0.39)	0.32 ± 0.19 (0.28 to 0.36)	0.125
Minimal vertical acromiohumeral distance (cm) Eab	0.36 ± 0.20 (0.32 to 0.40)	0.33 ± 0.20 (0.29 to 0.38)	0.361
Minimal vertical acromiohumeral distance (cm) Ead	0.37 ± 0.21 (0.33 to 0.42)	0.37 ± 0.21 (0.32 to 0.41)	0.947

The values of categorical variables were expressed by the number (percentage). The values of continuous variables were expressed by the mean and standard deviation (95% confidence interval of mean)

*Fab* full-can abduction phase, *Fad* full-can adduction phase, *Eab* empty-can abduction phase, *Ead* empty-can adduction phase

* *p* < 0.05

### Prediction of early treatment success

Among the 90 patients receiving dual-target injections, 70 (77.7%) experienced early treatment success. There was no significant difference between those with and without early treatment success in terms of gender, age, body weight/height, and body mass index. However, the group with early treatment success had less subscapularis tendon calcification and supraspinatus tendon full-thickness tears, more supraspinatus tendinopathy, and increased mVAHD in most postures except during the abduction phase in the full-can posture (Table 2). After adjusting for gender, age, and laterality of the painful sides, an enlarged minimal mVAHD during the adduction phase in the full-can posture, the abduction phase in the empty-can posture, and the adduction phase in the full-can posture was associated with early treatment success (Supplementary Table 2). The average area under the curve (AUC) for distinguishing between patients with and without early treatment success was 0.566 (95% CI, 0.417 to 0.415) for the full-can posture/abduction phase, 0.636 (95% CI, 0.485 to 0.787) for the full-can posture/adduction phase, 0.651 (95% CI, 0.513 to 0.790) for the empty-can posture/abduction phase, and 0.649 (95% CI, 0.522 to 0.775) for the empty-can posture/adduction phase (Supplementary Fig. 1). The mVAHD showed no significant difference between pre- and post-injection values, except in the adduction phase and full-can posture in the group without early treatment success (Supplementary Table 3).

**Table 2:** Baseline characteristics, static sonographic findings, and minimum vertical acromiohumeral distance in patients with and without early treatment success following dual-target injection

	Patients (*n* = 90)		
	Early treatment success (−) (*n* = 20)	Early treatment success (+) (*n* = 70)	*p*-value
Basic characteristics			
Female (*n*, %)	12 (60.0%)	44 (62.9%)	0.816
Age (years)	63.25 ± 9.32 (58.89 to 67.61)	58.86 ± 10.57 (56.34 to 61.38)	0.097
Height (cm)	160.39 ± 7.50 (156.54 to 164.25)	160.13 ± 7.92 (158.15 to 162.11)	0.663
Weight (kg)	59.71 ± 10.34 (54.39 to 65.03)	60.87 ± 11.26 (58.06 to 63.68)	0.821
Body mass index (kg/m^2^)	23.22 ± 3.79 (21.27 to 25.17)	23.63 ± 3.21 (22.83 to 24.43)	0.659
Laterality of painful shoulders (right side) (*n*, %)	9 (45.0%)	30 (42.9%)	0.865
Clinical findings			
Pain duration (months)	3.09 ± 1.83 (2.23 to 3.94)	3.57 ± 1.84 (3.12 to 4.02)	0.198
Resting pain before treatment (cm of VAS)	6.20 ± 1.06 (5.71 to 6.69)	6.29 ± 0.85 (6.08 to 6.49)	0.319
Night pain before treatment (cm of VAS)	6.05 ± 1.23 (5.47 to 6.63)	6.40 ± 0.95 (6.17 to 6.63)	0.090
Pain during overhead activities before treatment (cm of VAS)	6.65 ± 1.04 (6.16 to 7.14)	6.51 ± 1.06 (6.26 to 6.77)	0.835
Resting pain after treatment (cm of VAS)	4.94 ± 1.73 (4.13 to 5.75)	2.26 ± 0.83 (2.06 to 2.46)	< 0.001*
Night pain after treatment (cm of VAS)	4.80 ± 1.77 (3.97 to 5.63)	2.25 ± 0.88 (2.03 to 2.46)	< 0.001*
Pain during overhead activities after treatment (cm of VAS)	4.94 ± 1.92 (4.04 to 5.84)	2.23 ± 0.98 (1.99 to 2.47)	< 0.001*
Reduction in resting pain (cm of VAS)	−1.26 ± 1.58 (−2.00 to −0.52)	−3.98 ± 0.91 (−4.20 to −3.76)	< 0.001*
Reduction in night pain (cm of VAS)	−1.25 ± 1.55 (−1.97 to −0.52)	−4.12 ± 0.93 (−4.34 to −3.90)	< 0.001*
Reduction in pain during overhead activities (cm of VAS)	−1.71 ± 1.69 (−2.50 to −0.92)	−4.24 ± 1.09 (−4.50 to −3.97)	< 0.001*
Pain domain of SPADI before treatment	71.00 ± 18.92 (62.14 to 79.86)	73.83 ± 17.51 (69.65 to 78.00)	0.484
Function domain of SPADI before treatment	54.69 ± 27.73 (41.71 to 67.67)	55.74 ± 24.06 (50.00 to 61.47)	0.820
Total score of SPADI before treatment	60.92 ± 23.57 (49.89 to 71.95)	62.71 ± 20.09 (57.92 to 67.50)	0.752
Pain domain of SPADI after treatment	63.90 ± 17.75 (55.59 to 72.21)	26.29 ± 17.85 (21.97 to 30.61)	< 0.001*
Function domain of SPADI after treatment	54.79 ± 21.64 (44.66 to 64.91)	18.22 ± 16.59 (14.21 to 22.24)	< 0.001*
Total score of SPADI after treatment	58.25 ± 19.44 (49.16 to 67.35)	21.33 ± 16.29 (17.38 to 25.27)	< 0.001*
Static sonographic findings on the affected side			
Biceps tenosynovititis	6 (30.0%)	27 (38.6%)	0.483
Subscapularis calcification	5 (25.0%)	5 (7.1%)	0.025*
Subscapularis tear	0 (0.0%)	3 (4.3%)	0.346
Subscapularis tendinopathy	4 (20.0%)	11 (15.7%)	0.650
Supraspinatus calcification	2 (10.0%)	7 (10.0%)	1.000
Supraspinatus full-thickness tear	3 (15.0%)	2 (2.9%)	0.037
Supraspinatus partial tear	0 (0.0%)	6 (8.6%)	0.175
Supraspinatus tendinopathy	5 (25.0%)	35 (50.0%)	0.047*
Subdeltoid bursitis	4 (20.0%)	10 (14.3%)	0.534
Infraspinatus calcification	1 (5.0%)	4 (5.7%)	0.902
Infraspinatus tear	0 (0.0%)	0 (0.0%)	1.000
Infraspinatus tendinopathy	2 (10.0%)	5 (7.1%)	0.674
Minimal vertical acromiohumeral distance (cm) at baseline on the affected side			
Minimal vertical acromiohumeral distance (cm) in Fab	0.26 ± 0.18 (0.18 to 0.35)	0.30 ± 0.18 (0.26 to 0.35)	0.389
Minimal vertical acromiohumeral distance (cm) in Fad	0.24 ± 0.19 (0.15 to 0.33)	0.34 ± 0.19 (0.29 to 0.38)	0.046*
Minimal vertical acromiohumeral distance (cm) in Eab	0.25 ± 0.19 (0.16 to 0.34)	0.36 ± 0.20 (0.31 to 0.41)	0.034*
Minimal vertical acromiohumeral distance (cm) in Ead	0.28 ± 0.17 (0.20 to 0.36)	0.39 ± 0.22 (0.34 to 0.45)	0.032*

The values of categorical variables were expressed by the number (percentage). The values of continuous variables were expressed by the mean and standard deviation (95% confidence interval of mean)

*Fab* full-can abduction phase, *Fad* full-can adduction phase, *Eab* empty-can abduction phase, *Ead* empty-can adduction phase, *SPADI* shoulder pain and disability index, *VAS* visual analog scale

* *p* < 0.05

### Prediction of pain recurrence

Among the 70 patients with early treatment success, 25 (35.7%) experienced shoulder pain recurrence requiring repeated injections. The group with symptom recurrence had a greater body height and a more decreased mVAHD compared to those without recurrence (Table 3). After adjusting for gender, age, and laterality of the painful side, a decreased mVAHD across all phases and postures was associated with pain recurrence (Supplementary Table 4). The average area under the curve (AUC) for distinguishing between patients with and without pain recurrence was 0.715 (95% CI, 0.598 to 0.840) for the full-can posture/abduction phase, 0.727 (95% CI, 0.609 to 0.845) for the full-can posture/adduction phase, 0.700 (95% CI, 0.569 to 0.830) for the empty-can posture/abduction phase, and 0.658 (95% CI, 0.526 to 0.789) for the empty-can posture/adduction phase (Supplementary Fig. 2). There was no significant difference in mVAHD between pre- and post-injection values across various postures and shoulder movement phases, regardless of recurrence (Supplementary Table 5).

**Table 3:** Baseline characteristics, static sonographic findings, and minimal vertical acromiohumeral distance in patients without recurrent pain and with recurrent pain requiring repeated injections

	Patients with early success (*n* = 70)		
	Recurrence (−) (*n* = 45)	Recurrence (+) (*n* = 25)	*p*-value
Basic characteristics			
Female (*n*, %)	31 (68.9%)	13 (52.0%)	0.161
Age (years)	59.24 ± 11.30 (55.85 to 62.64)	58.16 ± 9.30 (54.32 to 62.00)	0.684
Height (cm)	158.94 ± 8.16 (156.36 to 161.51)	162.26 ± 7.15 (159.17 to 165.36)	0.045*
Weight (kg)	60.03 ± 11.17 (56.51 to 63.56)	62.36 ± 11.50 (57.38 to 67.33)	0.370
Body mass index (kg/m^2^)	23.64 ± 3.01 (22.69 to 24.59)	23.61 ± 3.60 (22.05 to 25.16)	0.973
Laterality of painful shoulders (right side) (*n*, %)	16 (35.6%)	14 (56.0%)	0.100
Clinical findings			
Pain duration (months)	3.68 ± 1.89 (3.11 to 4.24)	3.21 ± 1.76 (2.64 to 3.77)	0.296
Resting pain before treatment (cm of VAS)	6.40 ± 0.79 (6.17 to 6.63)	6.12 ± 0.99 (5.81 to 6.43)	0.080
Night pain before treatment (cm of VAS)	6.50 ± 0.85 (6.25 to 6.75)	6.12 ± 1.17 (5.75 to 6.48)	0.044*
Pain during overhead activities before treatment (cm of VAS)	6.65 ± 1.00 (6.36 to 6.94)	6.43 ± 1.11 (6.08 to 6.77)	0.363
Resting pain after treatment (cm of VAS)	2.38 ± 0.85 (2.13 to 2.63)	3.40 ± 1.97 (2.79 to 4.02)	0.044*
Night pain after treatment (cm of VAS)	2.38 ± 0.93 (2.10 to 2.66)	3.32 ± 1.94 (2.72 to 3.92)	0.059
Pain during overhead activities after treatment (cm of VAS)	2.37 ± 0.89 (2.11 to 2.64)	3.37 ± 2.15 (2.70 to 4.04)	0.083
Reduction in resting pain (cm of VAS)	−3.95 ± 0.89 (−4.21 to −3.68)	−2.71 ± 1.90 (−3.31 to −2.12)	0.003*
Reduction in night pain (cm of VAS)	−4.08 ± 0.82 (−4.32 to −3.83)	−2.80 ± 2.00 (−3.42 to −2.18)	0.003*
Reduction in pain during overhead activities (cm of VAS)	−4.22 ± 0.94 (−4.50 to −3.94)	−3.06 ± 1.99 (−3.68 to −2.44)	0.003*
Pain domain of SPADI before treatment	73.50 ± 16.86 (68.60 to 78.40)	72.86 ± 18.95 (66.95 to 78.76)	0.932
Function domain of SPADI before treatment	56.18 ± 23.90 (49.24 to 63.12)	54.73 ± 25.98 (46.64 to 62.83)	0.692
Total score of SPADI before treatment	62.86 ± 20.11 (57.02 to 68.70)	61.68 ± 21.76 (54.90 to 68.47)	0.722
Pain domain of SPADI after treatment	28.00 ± 18.02 (22.65 to 33.35)	42.33 ± 27.08 (33.89 to 50.77)	0.013*
Function domain of SPADI after treatment	20.22 ± 17.63 (14.98 to 25.45)	33.45 ± 27.12 (25.00 to 41.90)	0.031*
Total score of SPADI after treatment	23.21 ± 17.09 (18.13 to 28.29)	36.85 ± 26.50 (28.59 to 45.11)	0.018*
Static sonographic findings on the affected side			
Biceps tenosynovititis	13 (28.9%)	14 (56.0%)	0.026
Subscapularis calcification	4 (8.9%)	1 (4.0%)	0.447
Subscapularis tear	3 (6.7%)	0 (0.0%)	0.187
Subscapularis tendinopathy	7 (15.6%)	4 (16.0%)	0.961
Supraspinatus calcification	4 (8.9%)	3 (12.0%)	0.678
Supraspinatus full-thickness tear	2 (4.4%)	0 (0.0%)	0.285
Supraspinatus partial tear	3 (6.7%)	3 (12.0%)	0.445
Supraspinatus tendinopathy	24 (53.3%)	11 (44.0%)	0.454
Subdeltoid bursitis	5 (11.1%)	5 (20.0%)	0.309
Infraspinatus calcification	3 (6.7%)	1 (4.0%)	0.645
Infraspinatus tear	0 (0.0%)	0 (0.0%)	1.000
Infraspinatus tendinopathy	4 (8.9%)	1 (4.0%)	0.447
Minimal vertical acromiohumeral distance (cm) at baseline on the affected side			
Minimal vertical acromiohumeral distance (cm) in Fab	0.35 ± 0.18 (0.30 to 0.41)	0.21 ± 0.15 (0.15 to 0.28)	0.002*
Minimal vertical acromiohumeral distance (cm) in Fad	0.39 ± 0.20 (0.33 to 0.45)	0.24 ± 0.12 (0.19 to 0.29)	0.001*
Minimal vertical acromiohumeral distance (cm) in Eab	0.40 ± 0.20 (0.35 to 0.46)	0.27 ± 0.19 (0.19 to 0.35)	0.006*
Minimal vertical acromiohumeral distance (cm) in Ead	0.44 ± 0.22 (0.37 to 0.50)	0.32 ± 0.20 (0.24 to 0.40)	0.026*

The values of categorical variables were expressed by the number (percentage). The values of continuous variables were expressed by the mean and standard deviation (95% confidence interval of mean)

*Fab* full-can abduction phase, *Fad* full-can adduction phase, *Eab* empty-can abduction phase, *Ead* empty-can adduction phase, *SPADI* shoulder pain and disability index, *VAS* visual analog scale

* *p* < 0.05

### Comparison of pain recurrence between dual-target and standard subdeltoid-subacromial injection

**Fig. 4:**
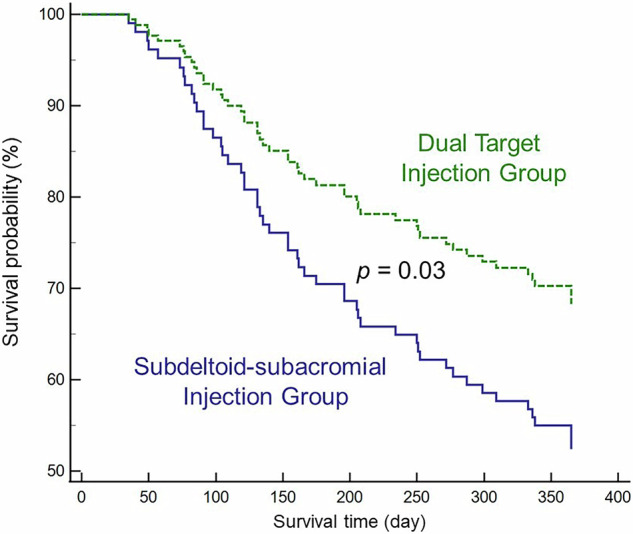
Survival analysis comparing time to pain recurrence between the group undergoing ultrasound-guided dual-target injection and the historical cohort receiving ultrasound-guided standard subdeltoid-subacromial injection

Ninety patients were retrospectively selected from a clinical registry of 164 individuals matched by age within a 5-year range, with no significant differences in age or sex distribution (Supplementary Table 6). The underlying sonographic pathologies of the affected shoulders of the 90 patients in the historical cohort are shown in Supplementary Table 7. Survival curve analysis showed a mean duration to pain recurrence of 309.1 ± 130.1 (95% CI, 286.7 to 331.4) days in the dual-target injection group compared to 267.5 ± 184.2 (95% CI, 235.8 to 299.2) days in the standard subdeltoid-subacromial injection group, with a statistically significant difference (*p* = 0.03) (Fig. 4).

## Discussion

Our study revealed several important findings. First, in patients with SIS, no significant difference in mVAHD was observed between the symptomatic and asymptomatic shoulders. Second, an increased mVAHD was associated with early treatment success, whereas a decreased mVAHD was linked to pain recurrence following dual-target injection. Third, patients who received dual-target injections experienced a longer pain-free period compared to those who underwent standard subdeltoid-subacromial injection.

Our study did not identify a significant difference in mVAHD between symptomatic and asymptomatic sides in patients with unilateral SIS. In contrast, Chang et al’s 2022 study [24] found a significantly smaller mVAHD in patients with SIS compared to the healthy population. We hypothesize that this discrepancy arises because Chang et al’s study included asymptomatic shoulders in the healthy population, while our study enrolled the symptom-free shoulder in the unilateral shoulder pain group. Eliason et al [25] found 73% of subacromial-subdeltoid bursitis present in both shoulders of patients with clinically diagnosed unilateral subacromial pain syndrome using ultrasound. Similarly, Barreto’s study [26] using magnetic resonance imaging showed no significant difference in the prevalence of various shoulder pathologies (except for full-thickness tears in the supraspinatus tendon and glenohumeral osteoarthritis) in patients with unilateral shoulder pain. We speculate that compensatory overuse of the asymptomatic shoulder or improper muscle tendinous biomechanics across both shoulders contributed to the non-significant difference in mVAHD between bilateral shoulders in our research.

Our findings expanded upon existing research by evaluating the dual-target injection technique beyond its immediate clinical benefits, incorporating long-term outcomes assessed through ultrasonographic subacromial motion metrics. Previous studies, such as those by Nidhi Agrawal et al [27] and Wang et al [8] and, primarily focused on procedural aspects and short-term clinical benefits of targeting both the subacromial-subdeltoid bursa and the long head of the biceps brachii tendon sheath, while our study provided novel insights into the biomechanical effects of this approach. Additionally, the randomized controlled trial conducted by Hou et al [28] demonstrated that corticosteroid injections into both targets were more effective than injection into the subacromial-subdeltoid bursa alone for managing hemiplegic shoulder pain. However, that study did not examine the impact of this technique on subacromial motion. By addressing these gaps, our study offered a more comprehensive perspective on how dual-target corticosteroid injections influenced both clinical outcomes and ultrasonographic findings, reinforcing the significance of this technique in optimizing long-term treatment strategies for shoulder pain.

In our study of patients receiving dual-target injections, an increase in mVAHD correlated with early treatment success, whereas a decrease in mVAHD corresponded with pain recurrence. Quantitative dynamic ultrasound subacromial motion metrics allow real-time assessment of the humeral head against the lateral acromion, especially during moments when impingement is likely to occur [24]. The quantification of mVAHD allows for a precise assessment of risk, as demonstrated by the ROC curve analysis in our study. A reduced mVAHD may result from a combination of intrinsic biomechanical factors (such as inadequate activation of the middle and lower trapezius muscles leading to insufficient scapular rotation) and extrinsic structural considerations (such as a downward-sloping curved acromion), which are often not adequately addressed by corticosteroid injections alone [29]. It is well established that the therapeutic benefits of corticosteroid injections arise from their anti-inflammatory and immunosuppressive properties [30]. Injecting corticosteroids into the subacromial bursa or the glenohumeral joint is more effective in treating pathologies secondary to impingement, such as subdeltoid bursitis or rotator cuff tendinitis, than in addressing underlying biomechanical or structural issues. This assertion is supported by the comparison between baseline and post-1-month outcomes of dual-target injections, which revealed mostly statistically nonsignificant differences. Consequently, our study not only aids in predicting treatment success and recurrence but also underscores the importance of evaluating certain biomechanical and structural factors, or determining whether the patient may need targeted physical therapy aimed at correcting their biomechanics.

In this study, we found that the group receiving dual-target injection had a longer symptom-free period compared to the historical cohort undergoing standard subdeltoid-subacromial injection. The dual-target approach, in addition to the traditional subacromial injection, directs the injectate to the glenohumeral joints, enhancing medication coverage for pathologies within the glenohumeral capsule [8]. Moreover, symptoms of SIS can resemble early adhesive capsulitis, which is characterized by fibrotic and inflammatory contracture of the rotator interval, capsule, and coracohumeral ligaments [31]. A recent meta-analysis of six randomized controlled trials confirmed that the anterior approach (through the rotator cuff interval) is superior to the posterior approach (through the posterior glenohumeral joint) in improving shoulder external rotation and abduction at 12 weeks post-injection [32]. This effectiveness may result from direct corticosteroid infiltration into the thickened, hypervascular coracohumeral ligament in adhesive capsulitis. Our study aligns with existing evidence supporting ultrasound-guided injections through the rotator cuff interval for possible coexisting adhesive capsulitis and targeting the subdeltoid-subacromial bursa for SIS.

The study has several limitations. First, the mVAHD from subacromial motion metrics was obtained through manual labeling of bony features frame by frame, a process that was both labor-intensive and time-consuming. With the development of a pilot deep learning model to delineate subacromial motion metrics [33], a validation study could determine whether artificial intelligence achieves the same level of precision as manual methods. Second, the study primarily focused on patients with SIS, although dual-target injection may also benefit those with adhesive capsulitis. Future research should investigate whether subacromial motion metrics can predict treatment outcomes in patients with adhesive capsulitis. Third, our study compared dual-target and standard ultrasound-guided subacromial-subdeltoid injections. The individual or combined use of suprascapular [34], axillary [35], and lateral pectoral nerve [36] blocks should be incorporated into future comparative studies to further enhance chronic shoulder pain management. Fourth, while the use of a historical cohort allowed for comparison of the long-term effects between dual-target injections and subdeltoid-subacromial injections, it also introduced significant risks of bias, including selection bias, information bias, temporal bias, and recall bias. Future studies should use a prospective cohort or randomized trial to minimize these biases and better assess treatment effects. Fifth, the selection of historical controls from approximately 7 years earlier than the case cohort was based on findings from our prior study [8], which demonstrated that dual-target subacromial corticosteroid injections resulted in a lower rate of pain recurrence compared to standard subacromial corticosteroid injections. As a result, the dual-target approach became the preferred technique, making it difficult to identify patients who received standard subacromial corticosteroid injections in the historical cohort after 2020.

## Conclusion

Early treatment success and recurrent pain after dual-target corticosteroid injection for SIS can be predicted using mVAHD from subacromial motion metrics via dynamic ultrasound imaging. Dual-target injection had a longer effect than standard injection. Future studies should assess mVAHD’s predictive value with deep learning-derived metrics and its effectiveness for adhesive capsulitis patients.

## Supplementary information

ELECTRONIC SUPPLEMENTARY MATERIAL

## Data Availability

The datasets used and/or analyzed during the current study are available from the corresponding author on reasonable request.

## Abbreviations

AUC: Average area under the curve
CI: Confidence intervals
mVAHD: Minimal vertical acromiohumeral distance
ROC: Receiver operating characteristic
SIS: Subacromial impingement syndrome
SPADI: Shoulder Pain and Disability Index
VAS: Visual analog scale
