# Supplementary material for: Predictive value of subacromial motion metrics for the effectiveness of ultrasound-guided dual-target injection: a longitudinal follow-up cohort trial
Source: Insights Imaging. 2025 Jul 1;16:145. doi: 10.1186/s13244-025-01989-5 (PMC12214097; doi:10.1186/s13244-025-01989-5)
Supplement: Supplementary file 1 — ELECTRONIC SUPPLEMENTARY MATERIAL [file 13244_2025_1989_MOESM1_ESM.zip › Supplemental Table 7 (baseline sonographic findings).docx]

| **Underlying Sonographic Pathologies** | **Number (percentage)** |
| --- | --- |
| Biceps tenosynovititis | 19 (21.11%) |
| Subscapularis calcification | 21 (23.33%) |
| Subscapularis tear | 5 (5.56%) |
| Subscapularis tendinopathy | 15 (16.67%) |
| Supraspinatus calcification | 22 (24.44%) |
| Supraspinatus full thickness tear | 12 (13.33%) |
| Supraspinatus partial tear | 2 (2.22%) |
| Supraspinatus tendinopathy | 44 (48.89%) |
| Subdeltoid bursitis | 20 (22.22%) |
| Infraspinatus calcification | 4 (4.44%) |
| Infraspinatus tear | 3 (3.33%) |
| Infraspinatus tendinopathy | 2 (2.22%) |

**Supplemental Table 7** Sonographic diagnoses for the affected shoulder of the 90 matched controls in the historical cohort
