# Supplementary material for: Predictive value of subacromial motion metrics for the effectiveness of ultrasound-guided dual-target injection: a longitudinal follow-up cohort trial
Source: Insights Imaging. 2025 Jul 1;16:145. doi: 10.1186/s13244-025-01989-5 (PMC12214097; doi:10.1186/s13244-025-01989-5)
Supplement: Supplementary file 1 — ELECTRONIC SUPPLEMENTARY MATERIAL [file 13244_2025_1989_MOESM1_ESM.zip › Supplemental Table 1 (Follow up window).docx]

**Supplemental Table 1**. Recruitment and follow-up time window for patients receiving dual target injection

| **Time Point** | **Event** | **Outcome Criteria** | **Next Step** |
| --- | --- | --- | --- |
| Injection day (Day 0) | Patients receive a dual-target injection | Not applicable | Proceed to follow-up |
| 4-Week Follow-Up (Day 28) | Assess pain relief using visual analogue scale (VAS) score | Responders of successful early outcome: VAS reduction >30% in any of three pain subdomains.  Non-responders: No significant improvement | No further structured follow-up for non-responders |
| Post 4-Week Follow-Up until final follow-Up (≤365 days post-injection) | No routine follow-ups unless symptoms recur.  Recurrence is defined as the need for a subsequent injection due to recurrent shoulder pain in initial responders. | - Recurrence: Patients who required additional treatment due to shoulder pain; their final follow-up is the day of recurrence (within 365 days).  - No recurrence: Patients without documented shoulder complaints requiring treatment within 365 days; their final follow-up is at 365 days post-injection. | Data recorded and classified as recurrence (follow-up period <365 days) or no recurrence (follow-up period = 365 days). |
