# Supplementary material for: Predictive value of subacromial motion metrics for the effectiveness of ultrasound-guided dual-target injection: a longitudinal follow-up cohort trial
Source: Insights Imaging. 2025 Jul 1;16:145. doi: 10.1186/s13244-025-01989-5 (PMC12214097; doi:10.1186/s13244-025-01989-5)
Supplement: Supplementary file 1 — ELECTRONIC SUPPLEMENTARY MATERIAL [file 13244_2025_1989_MOESM1_ESM.zip › Supplemental Table 2 (OR for early treatment success).docx]

**Supplemental Table 2.** Multivariate logistic regression analysis of the association between baseline minimal vertical acromiohumeral distance and early treatment success, adjusted for sex, age, and shoulder pain laterality

| **Variables** | **Odd ratio (95% CI)** | **p value** |
| --- | --- | --- |
| Minimal vertical acromiohumeral distance (cm) in Fab | 5.089 (0.257 to 100.715) | 0.285 |
| Minimal vertical acromiohumeral distance (cm) in Fad | 34.143 (1.455 to 801.185) | **0.028*** |
| Minimal vertical acromiohumeral distance (cm) in Eab | 25.184 (1.272 to 498.740) | **0.034*** |
| Minimal vertical acromiohumeral distance (cm) in Ead | 26.045 (1.492 to 454.592) | **0.026*** |

* Indicates *p* <0.05. Fab: full-can abduction phase; Fad: full-can adduction phase; Eab: empty-can abduction phase; Ead: empty-can adduction phase.
