# Supplementary material for: Predictive value of subacromial motion metrics for the effectiveness of ultrasound-guided dual-target injection: a longitudinal follow-up cohort trial
Source: Insights Imaging. 2025 Jul 1;16:145. doi: 10.1186/s13244-025-01989-5 (PMC12214097; doi:10.1186/s13244-025-01989-5)
Supplement: Supplementary file 1 — ELECTRONIC SUPPLEMENTARY MATERIAL [file 13244_2025_1989_MOESM1_ESM.zip › Supplemental Table 3 (Baseline and post-injection ).docx]

**Supplement Table 3.** Comparison of minimal vertical acromiohumeral distance between baseline and after injection in patients receiving dual-target injection

|  | **Without early success (n = 20)** | | |  |  | **With early success (n = 70)** | |  |
| --- | --- | --- | --- | --- | --- | --- | --- | --- |
|  | **Before injection** | **After injection** | **p value** |  |  | **Before injection** | **After injection** | **p value** |
| Minimal vertical acromiohumeral distance (cm) in Fab | 0.26 ± 0.18 (0.18 to 0.35) | 0.32 ± 0.17 (0.24 to 0.40) | 0.126 |  |  | 0.30 ± 0.18 (0.26 to 0.35) | 0.32 ± 0.17 (0.28 to 0.37) | 0.343 |
| Minimal vertical acromiohumeral distance (cm) in Fad | 0.24 ± 0.19 (0.15 to 0.33) | 0.34 ± 0.20 (0.25 to 0.44) | **0.044*** |  |  | 0.34 ± 0.19 (0.29 to 0.38) | 0.36 ± 0.18 (0.32 to 0.41) | 0.235 |
| Minimal vertical acromiohumeral distance (cm) in Eab | 0.25 ± 0.19 (0.16 to 0.34) | 0.27 ± 0.21 (0.17 to 0.36) | 0.852 |  |  | 0.36 ± 0.20 (0.31 to 0.41) | 0.37 ± 0.20 (0.32 to 0.41) | 0.605 |
| Minimal vertical acromiohumeral distance (cm) in Ead | 0.28 ± 0.17 (0.20 to 0.36) | 0.28 ± 0.23 (0.18 to 0.39) | 0.881 |  |  | 0.39 ± 0.22 (0.34 to 0.45) | 0.39 ± 0.20 (0.34 to 0.44) | 0.993 |

* Indicates *p* <0.05. The values of continuous variables were expressed by the mean and standard deviation (95% confidence interval of mean). Fab: full-can abduction phase; Fad: full-can adduction phase; Eab: empty-can abduction phase; Ead: empty-can adduction phase.
