# Supplementary material for: Predictive value of subacromial motion metrics for the effectiveness of ultrasound-guided dual-target injection: a longitudinal follow-up cohort trial
Source: Insights Imaging. 2025 Jul 1;16:145. doi: 10.1186/s13244-025-01989-5 (PMC12214097; doi:10.1186/s13244-025-01989-5)
Supplement: Supplementary file 1 — ELECTRONIC SUPPLEMENTARY MATERIAL [file 13244_2025_1989_MOESM1_ESM.zip › Supplemental Table 4 (HR for recurrence).docx]

**Supplemental Table 4.** Analysis using the Cox proportional hazards model was conducted to examine the association between minimal vertical acromiohumeral distance at baseline, adjusting for sex, age, and the laterality of the painful side.

| **Variables** | **Hazard ratio (95% CI)** | **p value** |
| --- | --- | --- |
| Minimal vertical acromiohumeral distance (cm) in Fab | 0.036 (0.002 to 0.567) | **0.018*** |
| Minimal vertical acromiohumeral distance (cm) in Fad | 0.032 (0.002 to 0.432) | **0.010*** |
| Minimal vertical acromiohumeral distance (cm) Eab | 0.015 (0.001 to 0.212) | **0.002*** |
| Minimal vertical acromiohumeral distance (cm) in Ead | 0.047 (0.004 to 0.523) | **0.013*** |

* Indicates *p* <0.05. Fab: full-can abduction phase; Fad: full-can adduction phase; Eab: empty-can abduction phase; Ead: empty-can adduction phase.
