# Supplementary material for: Predictive value of subacromial motion metrics for the effectiveness of ultrasound-guided dual-target injection: a longitudinal follow-up cohort trial
Source: Insights Imaging. 2025 Jul 1;16:145. doi: 10.1186/s13244-025-01989-5 (PMC12214097; doi:10.1186/s13244-025-01989-5)
Supplement: Supplementary file 1 — ELECTRONIC SUPPLEMENTARY MATERIAL [file 13244_2025_1989_MOESM1_ESM.zip › Supplemental Table 5 (post-injection for recurrence).docx]

**Supplemental Table 5.** Comparison of the minimal vertical acromiohumeral distance between patients with and without recurrence at baseline and after injection

|  | **Recurrence (-), n = 45** | | |  | **Recurrence (+), n = 25** | |  |
| --- | --- | --- | --- | --- | --- | --- | --- |
|  | **Before injection** | **After injection** | **p value** |  | **Before injection** | **After injection** | **p value** |
| Minimal vertical acromiohumeral distance (cm) in Fab | 0.35 ± 0.18 (0.30 to 0.41) | 0.37 ± 0.17 (0.32 to 0.42) | 0.470 |  | 0.21 ± 0.15 (0.15 to 0.28) | 0.23 ± 0.15 (0.17 to 0.29) | 0.581 |
| Minimal vertical acromiohumeral distance (cm) in Fad | 0.39 ± 0.20 (0.33 to 0.45) | 0.42 ± 0.18 (0.37 to 0.47) | 0.369 |  | 0.24 ± 0.12 (0.19 to 0.29) | 0.26 ± 0.15 (0.20 to 0.32) | 0.493 |
| Minimal vertical acromiohumeral distance (cm) in Eab | 0.40 ± 0.20 (0.35 to 0.46) | 0.42 ± 0.19 (0.36 to 0.48) | 0.546 |  | 0.27 ± 0.19 (0.19 to 0.35) | 0.27 ± 0.16 (0.20 to 0.33) | 0.737 |
| Minimal vertical acromiohumeral distance (cm) in Ead | 0.44 ± 0.22 (0.37 to 0.50) | 0.44 ± 0.19 (0.39 to 0.50) | 0.852 |  | 0.32 ± 0.20 (0.24 to 0.40) | 0.29 ± 0.17 (0.22 to 0.36) | 0.798 |

* Indicates *p* <0.05. The values of continuous variables were expressed by the mean and standard deviation (95% confidence interval of mean). Fab: full-can abduction phase; Fad: full-can adduction phase; Eab: empty-can abduction phase; Ead: empty-can adduction phase.
