# Supplementary material for: Predictive value of subacromial motion metrics for the effectiveness of ultrasound-guided dual-target injection: a longitudinal follow-up cohort trial
Source: Insights Imaging. 2025 Jul 1;16:145. doi: 10.1186/s13244-025-01989-5 (PMC12214097; doi:10.1186/s13244-025-01989-5)
Supplement: Supplementary file 1 — ELECTRONIC SUPPLEMENTARY MATERIAL [file 13244_2025_1989_MOESM1_ESM.zip › Supplemental Table 6 ( subacromial and dual target) (20250324).docx]

**Supplemental Table 6.** Baseline characteristics of the historical cohort receiving subdeltoid-subacromial injection compared to those of the group receiving dual-target injection

|  | **Patients receiving subdeltoid-acromial bursa injection  (n = 90)** | **Patients receiving dual-target injection (n = 90)** | **p value** |
| --- | --- | --- | --- |
| Basic characteristics |  |  |  |
| Female (n, %) | 51 (56.7%) | 56 (62.2%) | 0.448 |
| Age (years) | 58.80 ± 9.02 (56.91 to 60.69) | 59.83 ± 10.42 (57.65 to 62.02) | 0.478 |
| Body mass index (kg/m^2^) | 23.83 ± 4.12  (22.74 to 24.91) | 23.54 ± 3.32  (22.81 to 24.27) | 0.918 |
| Laterality of painful shoulders (right side) (n, %) | 58 (54.4%) | 39 (43.3%) | 0.004* |
| Years of recruitment | 2013/1/16 to 2015/9/23 | 2020/10/5 to 2022/11/24 | - |
| Pain duration (months) | 7.17 ± 14.70  (4.09 to 10.25) | 3.46 ± 1.84  (3.06 to 3.85) | 0.224 |
| Corticosteroid use | 40 mg of triamcinolone acetonide (entirely to the subdeltoid-subacromial bursa) | 40 mg of triamcinolone acetonide (half to the subdeltoid-subacromial bursa and the other half to the rotator interval) |  |
| Information source of recurrence | | | |
| Hospital visit / record | 82 (91.11%) | 71 (78.89%) | 0.022* |
| Telephone contact | 8 (8.89%) | 17 (18.89%) | 0.052 |
| Censor | 0 (0.00%) | 2 (2.22%) | 0.155 |

* Indicates *p* <0.05. The values of categorical variables were expressed by the number (percentage). The values of continuous variables were expressed by the mean and standard deviation (95% confidence interval of mean). Censored cases refer to individuals who showed no documented recurrence within 365 days after the injections, either through hospital visits or records, but whose status could not be confirmed via telephone contact.
