# Supplementary figures and images for: Predictive value of subacromial motion metrics for the effectiveness of ultrasound-guided dual-target injection: a longitudinal follow-up cohort trial
Source: Insights Imaging. 2025 Jul 1;16:145. doi: 10.1186/s13244-025-01989-5 (PMC12214097; doi:10.1186/s13244-025-01989-5)

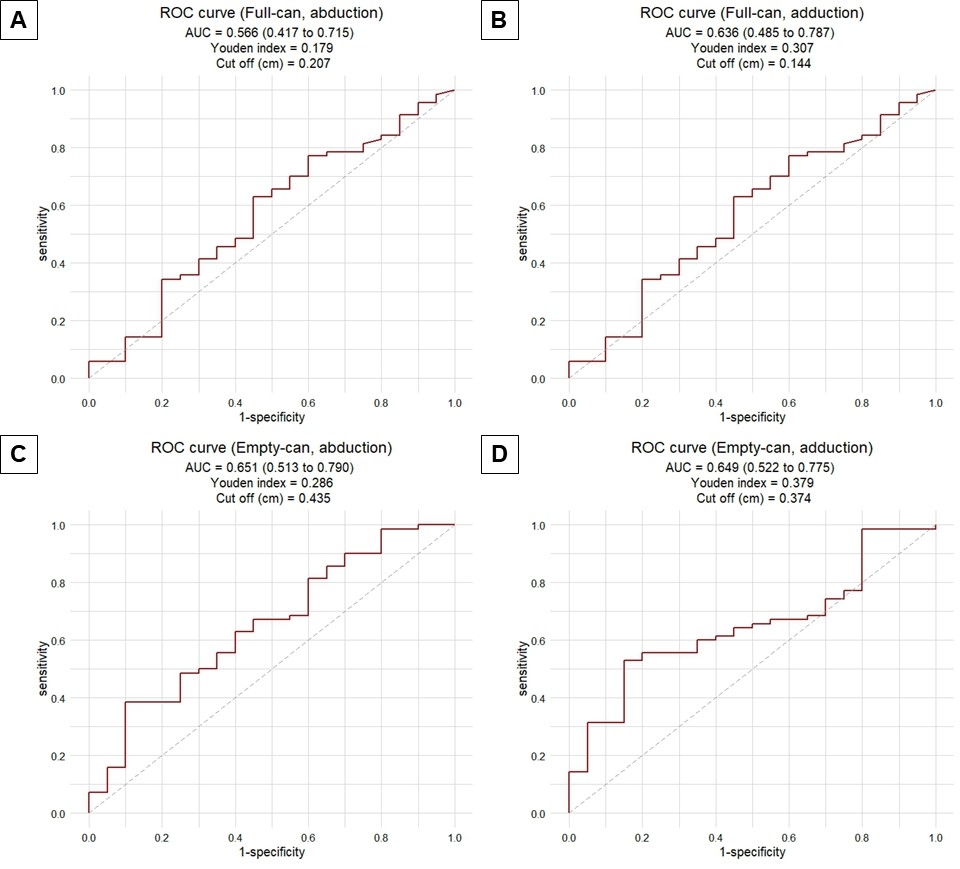

Supplement: Supplementary file 1 — ELECTRONIC SUPPLEMENTARY MATERIAL [file 13244_2025_1989_MOESM1_ESM.zip › Supplemental Figure 1.jpg]

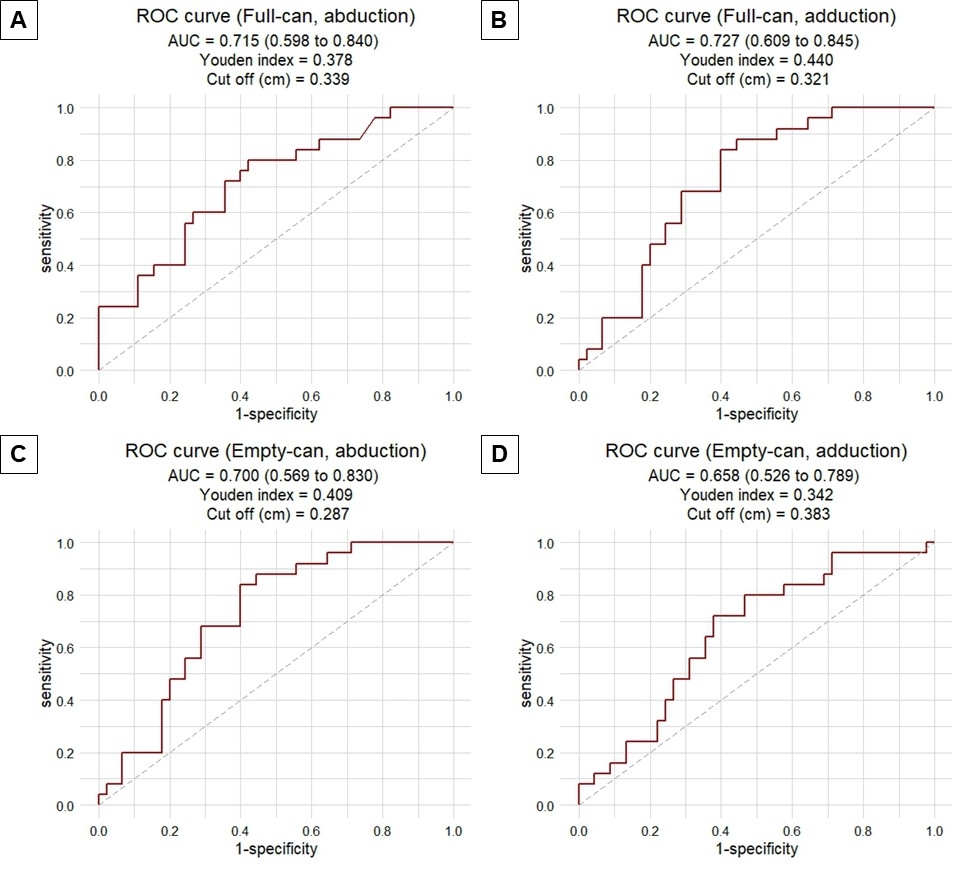

Supplement: Supplementary file 1 — ELECTRONIC SUPPLEMENTARY MATERIAL [file 13244_2025_1989_MOESM1_ESM.zip › Supplemental Figure 2.jpg]
